# Borneol promotes apoptosis of Human Glioma Cells through regulating HIF-1a expression via mTORC1/eIF4E pathway

**DOI:** 10.7150/jca.45304

**Published:** 2020-06-07

**Authors:** Zeng Wang, Qinglin Li, Liang Xia, Xia Li, Caixing Sun, Qiong Wang, Xinjun Cai, Guonong Yang

**Affiliations:** 1 Pharmacy Department, Institute of Cancer and Basic Medicine (ICBM), Chinese Academy of Sciences; Cancer Hospital of the University of Chinese Academy of Sciences; Zhejiang Cancer Hospital, Hangzhou 310022, People's Republic of China.; 2 Neurotumor surgery department, Institute of Cancer and Basic Medicine (ICBM), Chinese Academy of Sciences; Cancer Hospital of the University of Chinese Academy of Sciences; Zhejiang Cancer Hospital, Hangzhou 310022, People's Republic of China.; 3 Cancer Institute department, Institute of Cancer and Basic Medicine (ICBM), Chinese Academy of Sciences; Cancer Hospital of the University of Chinese Academy of Sciences; Zhejiang Cancer Hospital, Hangzhou 310022, People's Republic of China.; 4 Department of pharmacy, ZheJiang Chinese Medicine and Western Medicine Integrated Hospital, 310003, Hangzhou, Zhejiang, P. R. China.

**Keywords:** malignant glioma, borneol, HIF-1a, apoptosis

## Abstract

The main reason for the failure of malignant glioma treatment is local tumor recurrence. Tumor cells in hypoxic microenvironment activate HIF-1 α transcription, and thus promoting tumor invasion and metastasis is one of the important reasons. In our previous study, we clearly established that borneol opens the blood-brain tumor barrier and its related mechanism. However, the effects of borneol itself on glioma proliferation have not yet been elucidated. Therefore, in this study, we evaluated the effect of borneol on glioma by constructing *in vivo* SD rat brain glioma model and *in vitro* human primary cultured glioma cell model. We found that borneol could suppress the proliferation of primary glioma cells and the tumor volume of SD rat brain glioma. Further, we measured the apoptosis effect induced by borneol in human primary cultured glioma cells. The results showed that the higher the concentration of borneol, the higher the apoptosis rate of human primary cultured glioma cells, but the effect was reversed after transfection of HIF-1 overexpression plasmid; In addition, borneol could downregulate the expression of Bcl-2 and upregulation the expression of Bax and caspase-3, similarly, the effect was also reversed after transfection of HIF-1 overexpression plasmid, suggesting that the apoptosis effect induced by borneol in human primary cultured glioma cells is mediated via HIF-1α. Moreover, the bioinformatics analysis of correlation between HIF-1α and apoptosis-related factors based on CGGA database showed that there was a positive correlation between the expression of eIF4E and HIF-1 α (P < 0.05), and in patients with high expression of eIF4E and HIF-1α had poor survival and prognosis (P<0.001). It was further discovered that in the human primary cultured glioma cells borneol regulated HIF-1a expression via mTORC1/eIF4E pathway. In conclusion, the findings of the present study suggest that HIF-1α may be a key factor in borneol induced apoptosis of glioma cells, and mTORC1 / eIF4E pathway is involved in the HIF-1α regulation by borneol in malignant glioma. Our results not only reveal the target and molecular mechanism and action of borneol leading to promote apoptosis in glioma cells, but also provide experimental basis and theoretical support for the clinical application of borneol.

## Introduction

Glioma is the most common intracranial malignant tumor. It not only compresses normal brain tissues, increasing intracranial pressure and causing nervous system dysfunction, but also seriously threatens the health and life of patients 1. High-grade gliomas (WHO III and IV grade gliomas) have a high incidence, approximately accounting from 35% to 45% of primary intracranial malignancies worldwide 2, 3. Currently, the main clinical treatment of malignant gliomas is surgical, combined with radiotherapy and chemotherapy used to increase its efficacy, which is generally low.

The main reason for the failure of the treatment of malignant glioma is local tumor recurrence, which is closely related to the hypoxic microenvironment of malignant glioma. Tumor cells in a hypoxic microenvironment activated the transcription of hypoxia inducible factor-1α (HIF-1α), which was used as transcription factors promote tumor invasion, metastasis and resistance to radiotherapy and chemotherapy. In previous studies, the expression level of HIF-1α was found to be positively correlated with the pathological grade of glioma 4. The higher pathological grade of glioma was associated with faster tumor growth and infiltration, further promoting the transcription and translation of genes related to angiogenesis. The increased neovascularization provides more oxygen and other nutrients needed for peripheral tumor growth, thus promoting continuous tumor growth and invasion 5. Moreover, apoptosis impairment is also a hallmark of glioblastoma multiforme (GBM) 6 and is associated with tumor malignancy and poor prognosis 7,8. The role of HIF-1α in apoptosis process is complex. HIF-1α appears to have cell type-dependent pro- and antiapoptotic functions. Evidence has been highlighted that HIF-1α may inhibit apoptosis in GBM 9.

Studies at home and abroad have shown that the expression of HIF-1 α is regulated by many signaling pathways, among which the activation of mammalian rapamycin target (mTOR) related signaling pathway can increase the expression of HIF-1 α, maintain the acidic microenvironment of tumors, improve the invasion and metastasis of tumor cells, and lead to poor prognosis of solid tumors 10, 11. mTOR is a well conserved tyrosine kinase with a key role in the regulation of cell proliferation, growth, survival, division, movement, and angiogenesis 12. mTOR is subdivided into two complex subtypes, namely mTOR C1 and mTOR C2, where mTOR C1 is regulated by growth factors and nutrients to perform its function of governing cell growth and proliferation 13, 14. Eukaryotic translation initiation factor 4E (eIF4E) is considered an important surviving protein involved in the cell cycle, cell transformation, and other vital processes; of note, mTORC1 accelerated the synthesis of some oncogene proteins by activating the function of eIF4E 15. A previous study established that the increased expression of eIF4E increased the activity of cyclin D1, HIF-1α, and vascular endothelial growth factor; moreover, it enhanced mRNA translation and regulated cell proliferation and oxygen metabolism 16.

Borneolum Syntheticum is an antidote commonly used against fainting and convulsions 17. In addition, borneol was found to easily cross the blood-brain barrier, and some anti-tumor drugs, such as arsenic trioxide, nimustine, methotrexate, and cisplatin, promoted its penetration through the blood-brain tumor barrier 18-20. Furthermore, borneol has been used as a medicine since ancient times and is now also prescribed for brain disorders. It was shown to improve the energy metabolism in the area of the disorder and to protect the brain tissue from ischemic damage 21, 22.

Our research team has previously established intracranial transplantation tumor models of glioma C6, RG2, and 9L rats. We comprehensively studied the role and mechanism of action of borneol in opening the blood-brain tumor barrier^25^. Besides, the research of borneol has been reported mainly in three aspects, first, to study the effect of strengthening related treatment through blood-brain barrier); second, borneol itself as a carrier to transport drugs into brain tissue to play a role; third, borneol can cooperate with chemotherapy drugs by activating ROS mediated oxidative damage. Nevertheless, the effect of borneol on glioblastoma growth and HIF-1a expression are still unclear. Therefore, the purpose of this study was to investigate the effect of apoptosis induced by borneol in human malignant glioma primary cells, and the effect and relation of borneol on regulation of the mTORC1/eIF4E/HIF-1a signaling pathway. Better understanding the involvement of borneol in the target and molecular mechanism of apoptosis will promote its clinical application for the treatment of gliomas.

## Methods

### Construction of SD rat brain C6 glioma transplantation model

SPF male SD rats weighing 250-300g were provided by Shanghai sipur bikai experimental animal Co., Ltd. Before the operation, the animals were tested according to the routine. After confirming that there was no abnormal rotation behavior, they were anesthetized by intraperitoneal injection of 3% Pentobarbital Sodium 50mg / kg. Then the rats were fixed on the brain stereotaxic instrument, the hair was removed from the head and disinfected. Under aseptic condition, the skin of the cranial crest was cut along the median line, the periosteum was peeled off, and the anterior fontanelle was exposed. According to the atlas of brain stereotaxic localization wrote by Bao Xinmin et al, the right caudate nucleus was determined: 1 mm behind the anterior fontanelle, 3.0 mm to the right of the median line and 5.0 mm under the dura. The skull was drilled carefully in the required area of the operation, and 15ul cell suspension was injected into the right caudate nucleus of the rat by microinjector. The injection time is 10 minutes, keep the needle for 5 minutes, and then exit the microinjector slowly. After the operation, the skull hole was filled with bone wax, the incision skin was sutured. After the animals were awake, they were put back into the feeding cage.

### Animal grouping

After the establishment of the model, the rats were kept in SPF environment for 2 weeks to establish a stable and reliable transplanted tumor model. The rats were divided into 4 groups. A: Model group; B: borneol low dose group (4mg / kg); C: borneol medium dose group (8mg / kg); D: borneol high dose group (16mg / kg).

Rats inoculated with C6 glioma cells for 2 weeks were given borneol for 10 days. During the administration period, the general condition and survival period of each group of animals were recorded, and the animals' foraging, drinking water and limb movement were observed. The rats were killed and the glioma tissue was removed for subsequent experimental research.

### The expression of HIF-1α, mTORC1 and eIF4E protein in C6 rat model of intracranial transplantation tumor treated by borneol was observed by immunohistochemistry

The brain tissue of the rats was fixed with 10% neutral formaldehyde, cut along the injection site, and then embedded into slices. Paraffin sections were dried at 60 °C for 1 hour, dewaxed by xylene and graded alcohol (100% 3min, 100% 3min, 95% 3min, 90% 3min, 85% 3min, 75% 3min, distilled water 3min, distilled water 3min, PBS 3min). Antigen repair: place the section in 0.1mol/l citric acid solution with pH = 6.0, use 100 fire power of microwave oven for 3 minutes to slightly boil, use 50 fire power for 7 minutes, stop heating and then naturally cool for 20-30 minutes. Rinse with PBS for 5min × 3 / time. Immunohybridization: incubation with 3% H2O2 for 10 minutes to eliminate endogenous peroxidase activity; PBS washing, 5 minutes × 3 times. Add 5% BSA to the closed solution, and keep it in a wet box for 30 minutes. Wipe off the sealing liquid with filter paper, do not wash. Drop the first antibody of appropriate concentration (1:50 in average) and incubate it overnight in a wet box at 4 °C, then wash the first antibody with PBS for 5 minutes × 3 times, and wipe off PBS outside the sample with filter paper. Drop the biotinylated second antibody working solution, incubate in a wet box at room temperature for 20 minutes, wash the second antibody with PBS for 5 minutes × 3 times, and wipe off PBS outside the sample with filter paper. Drop the working solution of streptavidin labeled with horseradish enzyme, incubate it in a wet box at room temperature for 20 minutes, wash it with PBS for 5 minutes × 3 times, and wipe off PBS outside the sample with filter paper. DAB chromogenic agent and tap water should be fully rinsed. Then the hematoxylin was re dyed, dehydrated, transparent and neutral resin sealed. The tumor tissues of rats were randomly selected under 10 × 40 times microscope.

### Construction of an *in vitro* model of primary cultured glioma cells

Fresh human glioma tissue pieces were rinsed with serum-free culture medium and cut into small pieces of lmm 3 in size. 0.25% trypsin, digested in 37 °C water bath for 30 minutes, and filtered through a 100-mesh steel mesh. Transfer to a centrifuge tube and centrifuge at 1500 to 2000 rpm for 10 minutes, then remove the supernatant. The pellet was resuspended in a culture solution containing no calf serum, centrifuged twice at the same speed and the same time, and the supernatant was removed. Cells were resuspended in 1640 medium containing 10% A and bovine serum count. 5 × 10^5^ cells per well were transplanted into a 96 culture plate, and all cells were cultured in a humid environment at 37 ° C and 5% CO_2_. The growing cells were collected and observed under a microscope and a laser confocal microscope, and compared with the results of the original surgical pathological examination to confirm that the growing cells were human glioma cells. This study was approved by the Medical Ethical Committee of Zhejiang Cancer Hospital.

### Construction of HIF-1α gene overexpression primary glioma cells

Construct the expression plasmid of HIF-1α sequence, and use the Opti-MEM (Invitrogen) and Lipofectmine 3000 (Invitrogen) to insert the plasmid containing HIF-1α cDNA into the primary glioma cells by liposome transfection according to the instructions to construct HIF -1α Overexpressing Primary Glioma Cell Model.

### Construction of mTORC1 gene silencing and eIF4E gene silencing primary glioma cells

Design and synthesize the siRNA sequence of mTORC1. According to the product instructions, use Opti-MEM (Invitrogen) and Oligofectamine (Invitrogen) to transfect siRNA into primary glioma cells and silence the mTORC1 gene in primary glioma cells.

Design and synthesize the eIF4E siRNA sequence. Follow the product instructions to use Opti-MEM (Invitrogen) and Oligofectamine (Invitrogen) to transfect siRNA into primary glioma cells, and silence the eIF4E gene in primary glioma cells.

### Drug treatment and cell proliferation test (CCK-8)

Simulate hypoxic conditions (1% O_2_), take human glioma primary cell suspension in logarithmic growth phase and inoculate it into a 96-well plate, and place the culture plate in the incubator for 24 hours; according to the normal group, The solvent control group (PBS), 10 μg / ml borneol group, 20 μg / ml borneol group, 40 μg / ml borneol group, 80 μg / ml borneol group were administered in groups for experiments, and 5Gy radiation dose X-rays were given. After 48 hours of administration, 10 μL of CCK-8 solution was added to each well and incubated in an incubator. The absorbance at 450 nm was measured with a microplate reader, and the cell survival rate was calculated. Cell activity = (A value of the experimental group-blank zero A value) / (control group A value-blank zero A value) × 100%. Three replicates of each group of cells were measured in parallel.

### Immunofluorescence detection

Take mTORC1 gene silencing and eIF4E gene silencing primary glioma primary cells, culture them for 48 hours under hypoxic conditions (1% O2), collect cell culture fluids, put them into sterile high-cleanness coverslips, and then take 60- 70% cell density seed plate, add 1-2mL of pre-chilled 4% paraformaldehyde (PFA) to each well after attaching the cells, fix for 10min and aspirate, rinse 3 times in PBS for 5min each time; add 1mL-2mL 0.5 per well % Triton X-100, aspirate for about 2min, add 1-2mL PBS for rinsing, aspirate PBS and rinse 3 times for 5min each time; block with appropriate blocking solution for 0.5h (blocking solution: 3% BSA); incubate the primary antibody Rinse 3 times in PBS for 5 min each; incubate secondary antibodies: add 80 μL of secondary antibody to each cover slip (the dilution ratio of the secondary antibody is diluted with 5% goat serum according to the instructions, generally 1: 500), and incubated at room temperature for 0.5 h in the dark Remove the secondary antibody (Note: All steps after the secondary antibody step need to be protected from light), rinse PBS twice for 5min each time; nuclear staining: add nuclear staining reagent (DAPI, generally 1-2min can be used, depending on the specific As the case may be). After staining, remove the nucleating reagent, rinse once with PBS, and then add 1 mL of PBS to keep it wet. Preparation: First add an appropriate amount of mounting media (Mounting media, anti-fluorescence quenching blocking tablets, usually placed on -20 °C), use a curved needle to lift the coverslip with the side of the cell facing down, shake the slide slightly to dry it, and then cover the adhered slide containing the quencher. Squeeze to the other side and suck excess mounting media away with absorbent paper. Coverslip: Capillary drops of nail polish are placed on the four corners of the slide for covertling, and protected from light and dried. Finally, ZEISS was observed and photographed under an inverted fluorescence microscope.

### Real-time PCR detection

The primary glioma cells of mTORC1 gene silencing and eIF4E gene silencing were taken and cultured for 48 h under hypoxic conditions (1% O2). Cell culture fluids were collected and the cells were lysed to extract total RNA. The cells in the cell culture dish were washed twice with 1 mL of PBS, added with 1 ml of Trizol solution, pipetted and mixed, and aspirated into a 1.5 mL EP tube to fully lyse the cells, and allowed to stand at room temperature for 5 min. Place in a clean bench, incubate for 5 min, 12000 rpm, and centrifuge for 10 min. Aspirate the supernatant in a new 1.5 mL centrifuge tube, add 200 μL of chloroform, shake well, and let stand at room temperature for 2 minutes, 4 °C, 12000 rpm, and centrifuge for 10 minutes. Aspirate the supernatant in a new 1.5 mL centrifuge tube, add 600 μL of isopropanol, mix well, leave it at room temperature for 15 min, 4 °C, 12000 rpm, centrifuge for 15 min, and discard the supernatant. Add 1 mL of 75% absolute ethanol (750 μL of absolute ethanol and 250 μL of DEPC water) to rinse the pellet, centrifuge at 12,000 rpm at 4 °C for 5 minutes, and discard the supernatant. Add 1 mL of absolute ethanol, rinse the pellet, centrifuge at 12,000 rpm at 4 °C for 5 min, discard the supernatant, and dry at room temperature for 10 min. Add 40 μL of DEPC water to dissolve RNA and store in -80 °C refrigerator for future use. The following reaction system was prepared for reverse transcription reaction. Reaction conditions: 42 °C, 15 min; 85 °C, 5 min. Prepare the following reaction system for real-time PCR reaction. Thoroughly mix the solution in the tube with a vortex shaker and briefly centrifuge at low speed. Reaction conditions: 95 °C, 10min denaturation; 95 °C, 15s; 60 °C, 60s; 40 cycles. The expression of eIF4E or HIF-1α were detected by real-time PCR.

The primary glioma cells were taken and cultured under hypoxic conditions (1% O2). After four different concentrations (10, 20, 40, 80 μ g / ml) of borneol were used to treat cells for 24 hours, mRNA was extracted from cells of each group, and the expression of HIF-1 α, mTORC1, eIF4E, Bcl-2, Bax and caspase-3 were detected by real-time PCR.

### Western blot detection

The primary glioma cells of mTORC1 gene silencing and eIF4E gene silencing were taken and cultured for 48 h under hypoxic conditions (1% O2). Cell culture fluids were collected and lyse the cells to extract the total protein, use the BCA method to measure and balance the protein concentration, isolate the protein by 10% SDS-PAGE, transfer the protein to the PVDF membrane by semi-dry electrophoresis, and block at room temperature for 2 h. The primary antibody was incubated overnight, washed, and the secondary antibody was incubated for 1 h. The ECL method was used to develop the color and the tablet was exposed. The gel imager analysis system was used to scan the western blot detection band for gray value. The protein expression of eIF4E or HIF-1 α were detected.

Primary human glioma cells were taken under hypoxic conditions (1% O2), after 4 different concentrations (10, 20, 40, 80 μg/ml) intervention for 24 hours, collect the cell culture solution, lyse the cells to extract the total protein. And the protein expression of HIF-1 α, mTORC1, eIF4E, p-eIF4E, Bcl-2, Bax and caspase-3 were detected.

The HIF -1α overexpressing primary glioma cells were taken under hypoxic conditions (1% O2), after borneol 40μg/ml intervention for 24 hours, collect the cell culture solution, lyse the cells to extract the total protein, use the BCA method to measure and balance the protein concentration, isolate the protein by 10% SDS-PAGE, transfer the protein to the PVDF membrane by semi-dry electrophoresis, and block at room temperature for 2 h. The primary antibody was incubated overnight, washed, and the secondary antibody was incubated for 1 h. The ECL method was used to develop the color and the tablet was exposed. The gel imager analysis system was used to scan the western blot detection band for gray value. The protein expression of Bcl-2, Bax and caspase-3 was detected.

### Detection of apoptosis rate by flow cytometry

After treated with 4 different concentrations (10, 20, 40, 80 μg/ml) of borneol for 24 hours, apoptosis was detected by flow cytometry. The human glioma cells in the logarithmic growth stage were implanted in the 6-well plate with a volume of 2ml per well, and the cell inoculation density was 1.2 × 10^6^ cells per well. After transfection, the cells were collected and washed twice by precooling PBS, and the cell concentration was adjusted to 1 × 10^6^ cells / ml. Add 500 μl of binding buffer, centrifugate the supernatant, add 100 μl of binding buffer and mix well, respectively add 5 μl of annexin V-FITC and 10 μl of PI, mix well, and react at room temperature without light for 15 minutes. Finally, 400 μl of the binding buffer was added and the apoptotic rate was detected by flow cytometry. The experiment was repeated three times.

After treated with 40 μg/ml borneol for 24 hours, apoptosis was detected by flow cytometry. The HIF -1α overexpressing primary glioma cells in the logarithmic growth stage were implanted in the 6-well plate with a volume of 2ml per well, and the cell inoculation density was 1.2 × 10^6^ cells per well. After transfection, the cells were collected and washed twice by precooling PBS, and the cell concentration was adjusted to 1 × 10^6^ cells / ml. Add 500 μl of binding buffer, centrifugate the supernatant, add 100 μl of binding buffer and mix well, respectively add 5 μl of annexin V-FITC and 10 μl of PI, mix well, and react at room temperature without light for 15 minutes. Finally, 400 μl of the binding buffer was added and the apoptotic rate was detected by flow cytometry. The experiment was repeated three times.

### Bioinformatics analysis

We utilised Chinese Glioma Genome Atlas (CGGA) database, to extract the expression data of HIF-1a and apoptosis-related genes in GBM, then based on these expression profile data, Pierce correlation analysis was carried out by using R software, finally, prognostic analysis was performed.

### Statistical Analysis

SPSS 22.0 statistical software was used for statistical analysis. All data were expressed as mean ± standard deviation (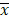
±s). Comparison between two samples was performed by t test. The survival curve was drawn by Kaplan-Meier method with R3.6 software and Log-rank test was conducted. The correlation analysis of gene expression was performed by Pearson method. P <0.05 was considered significant difference.

## Results

### Effect of borneol on the growth of brain glioma tissue in SD rats

It can be seen from Table 1 that compared with the model group, the tumor volume of borneol group is significantly smaller, and the higher the dose of borneol, the better the curative effect (P < 0.05 or P < 0.01).

### Effect of borneol on the expression of HIF-1α, mTORC1 and eIF4E in glioma tissue

**Figure 1:**
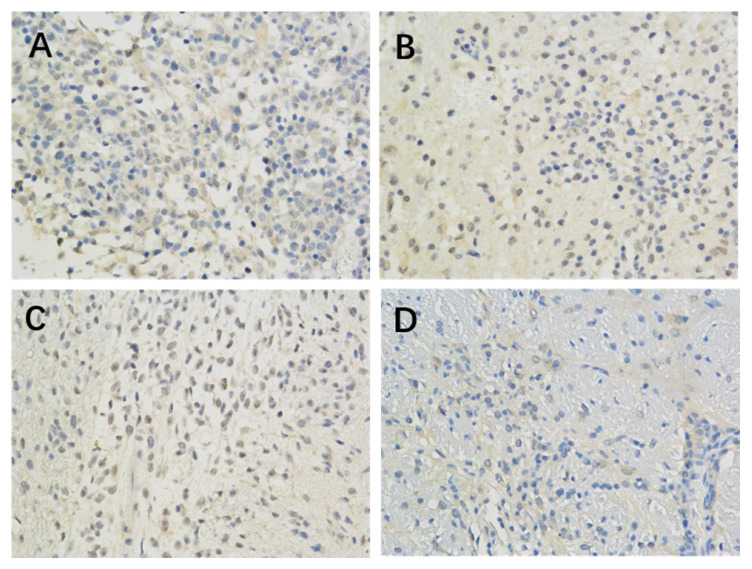
The effect of Borneol on the expression of HIF-1 protein in C6 rat brain glioma model (400-fold) A. Model Group (400 x) B. Low dose group of Borneol (400 x) C. Medium dose group of Borneol (400 x) D. High dose Group of BORNEOL (400 x).

**Figure 2:**
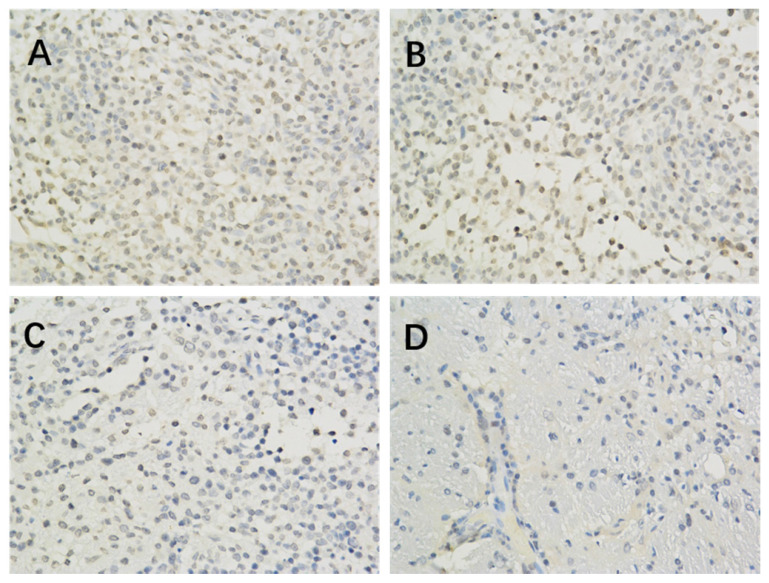
The effect of Borneol on the expression of MTORC1 protein (400-fold) in C6 rat intracranial transplanted glioma Model A. Model Group (400 x) B. Low dose group of Borneol (400 x) C. Medium dose group of Borneol (400 x) D. High dose Group of BORNEOL (400 x).

**Figure 3:**
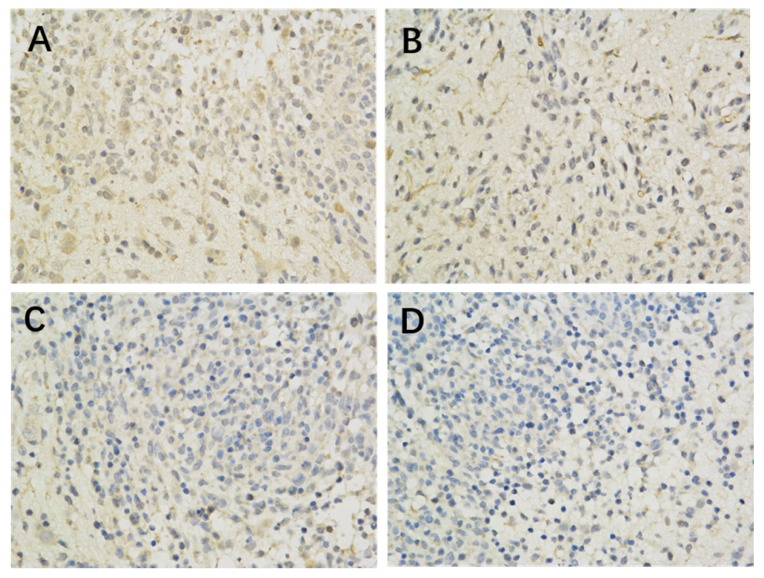
The effect of Borneol on the expression of eIF4E protein in the C6 rat model of intracranial transplanted Glioma (400-fold) A. Model Group (400 x) B. Low dose group of Borneol (400 x) C. Medium dose group of Borneol (400 x) D. High dose of Borneol (400 x).

The results of immunohistochemistry showed that HIF-1 α, mTORC1 and eIF4E were all expressed in the brain of each group. Compared with the model control group, the positive expression of HIF-1 α and eIF4E in each group was significantly lower (P < 0.05 or P < 0.01) (Figure 1, Table 2; Figure 2, Table 4). Compared with the model group, the positive expression of mTORC1 protein in each group was decreased, but only the middle dose group and high dose group of borneol had significant difference (P < 0.05 or P < 0.01) (Figure 3, Table 3).

### Separation of human primary glioma cells

**Figure 4:**
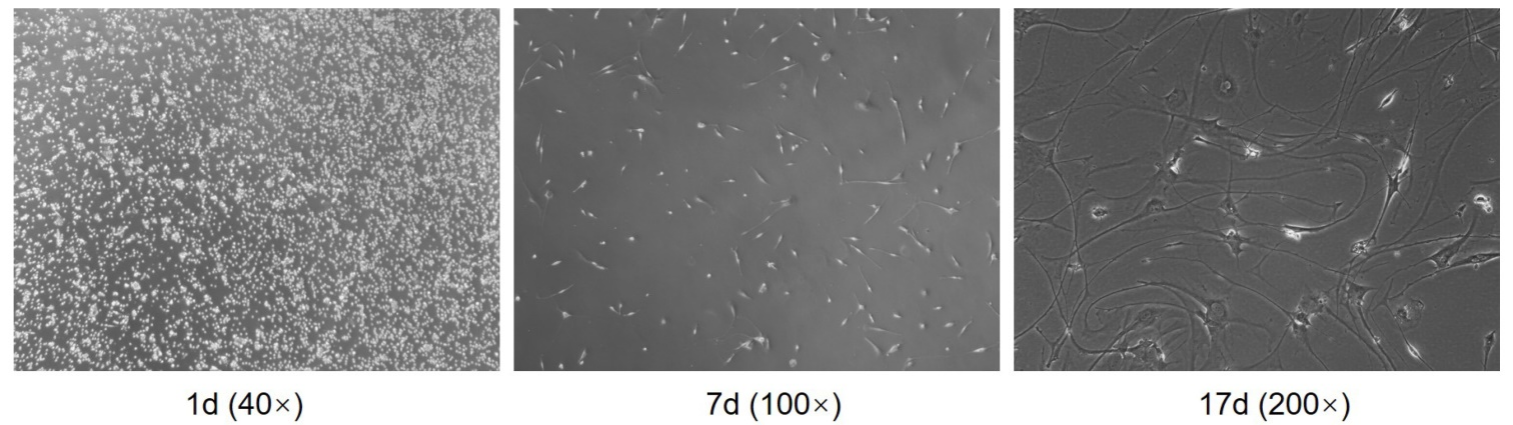
Separation of human primary glioma cells.

After digestion by trypsin, the cells of the glioma were initially rounded and dispersed in the culture medium. After 4 hours of culture in the cell suspension, the cells adhered to the wall and showed oval shape. Some cells formed colonies. After 24 hours, the cells expanded. After 48 hours, the number of cells was small and the density was low. However, the proliferation of cells was vigorous. The tumor cells varied in shape and size, mainly fusiform, and the body extended upward Protrude and stretch around. The primary glioma cells cultured successfully proliferated rapidly, and the cells after subculture grew vigorously, in good condition and stable character (Figure 4).

### Effect of borneol on the growth of primary cultured human glioma cells

**Figure 5:**
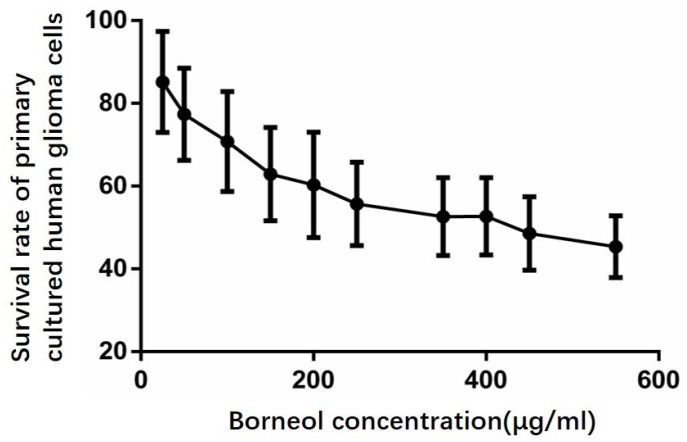
Effect of borneol on the growth of primary cultured human glioma cells.

According to Figure 5, compared with the control group, the cell survival rate of the treatment group has a downward trend, when the concentration of borneol reaches 40μg/ml or above, it shows a significant difference (P<0.05).

### Apoptosis detection in primary cultured human glioma cells

**Figure 6:**
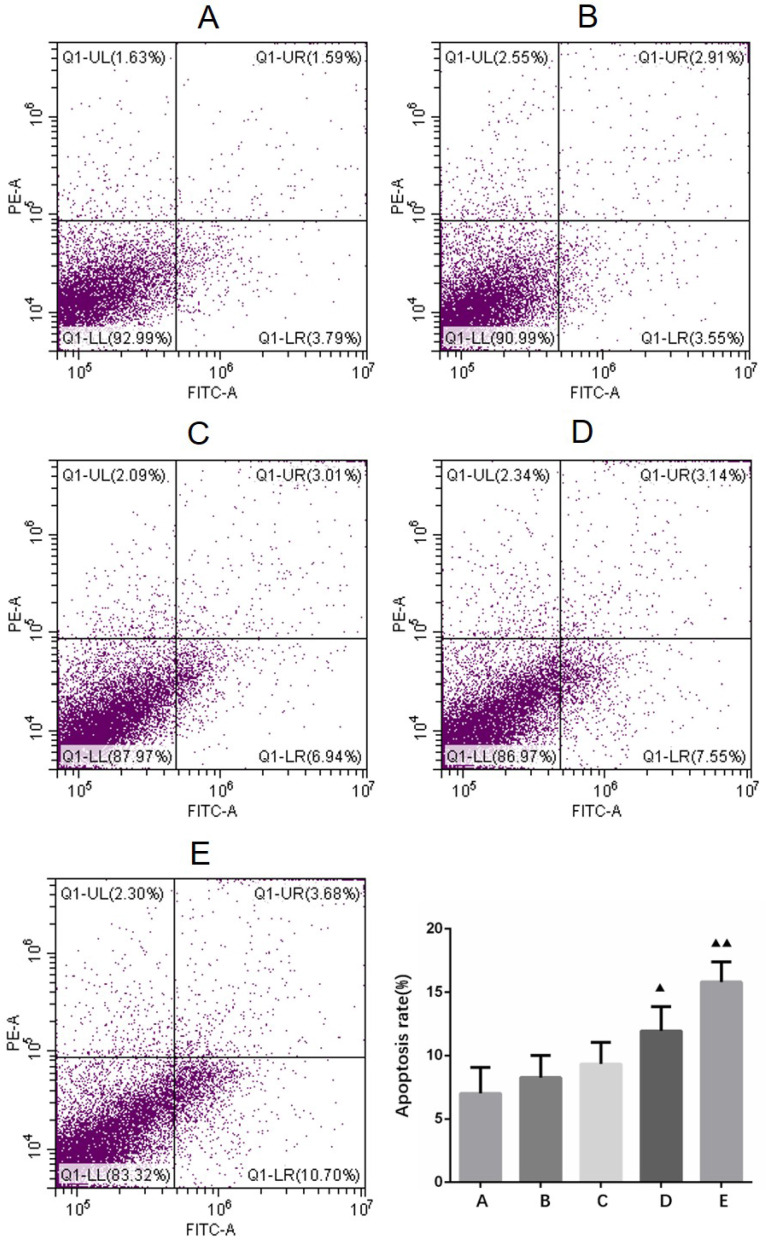
Apoptosis detection in primary cultured human glioma cells. A. Control B. Borneol 10 µg/ml C. Borneol 20 µg/ml D. Borneol 40 µg/ml E. Borneol 80 µg/ml comparison with the control group, ^▲^ P<0.05, ^▲▲^ P <0.01.

After the cells were treated with 4 different concentrations (10, 20, 40, 80 μg/ml) of borneol for 24 hours, the apoptosis rate showed an upward trend (8.27% - 15.81%), and there was a concentration dependence. The higher the concentration of borneol, the greater the apoptosis rate (Figure 6). When the borneol concentration reached 40 μg/ml, there was a significant difference between the two groups (P < 0.05).

### Effect of Borneol on apoptosis rate in Human Glioma Cells Transfected with HIF-1α Overexpression Plasmid

**Figure 7:**
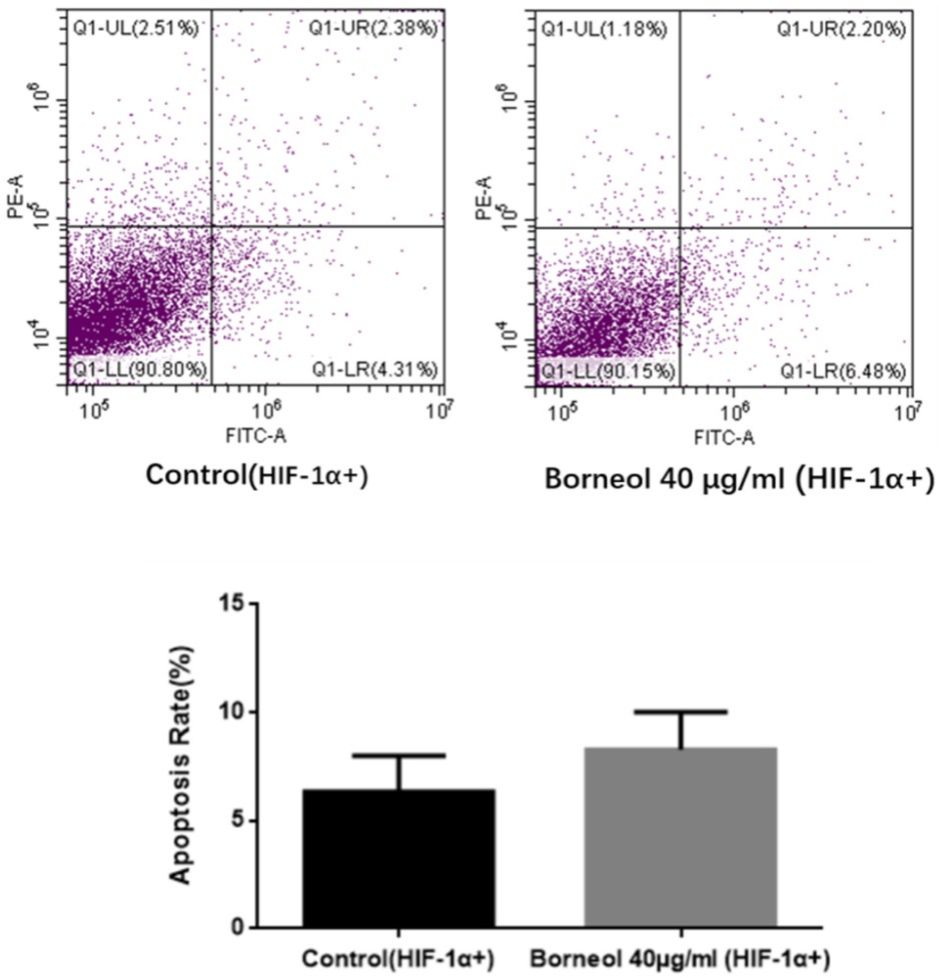
Apoptosis detection in primary cultured human glioma cells transfected with HIF-1α Overexpression Plasmid.

As shown in Figure 7, compared with the control group, the apoptosis rate of borneol 40 μg/ml had no significant difference (P > 0.05). The above results indicate that HIF-1α overexpression can hinder the apoptosis effect of borneol.

### The effect of Borneol on the expression of HIF-1α, mTORC1, eIF4E, Bcl-2, Bax and Caspase-3 in human glioma cells by qRT-PCR

**Figure 8:**
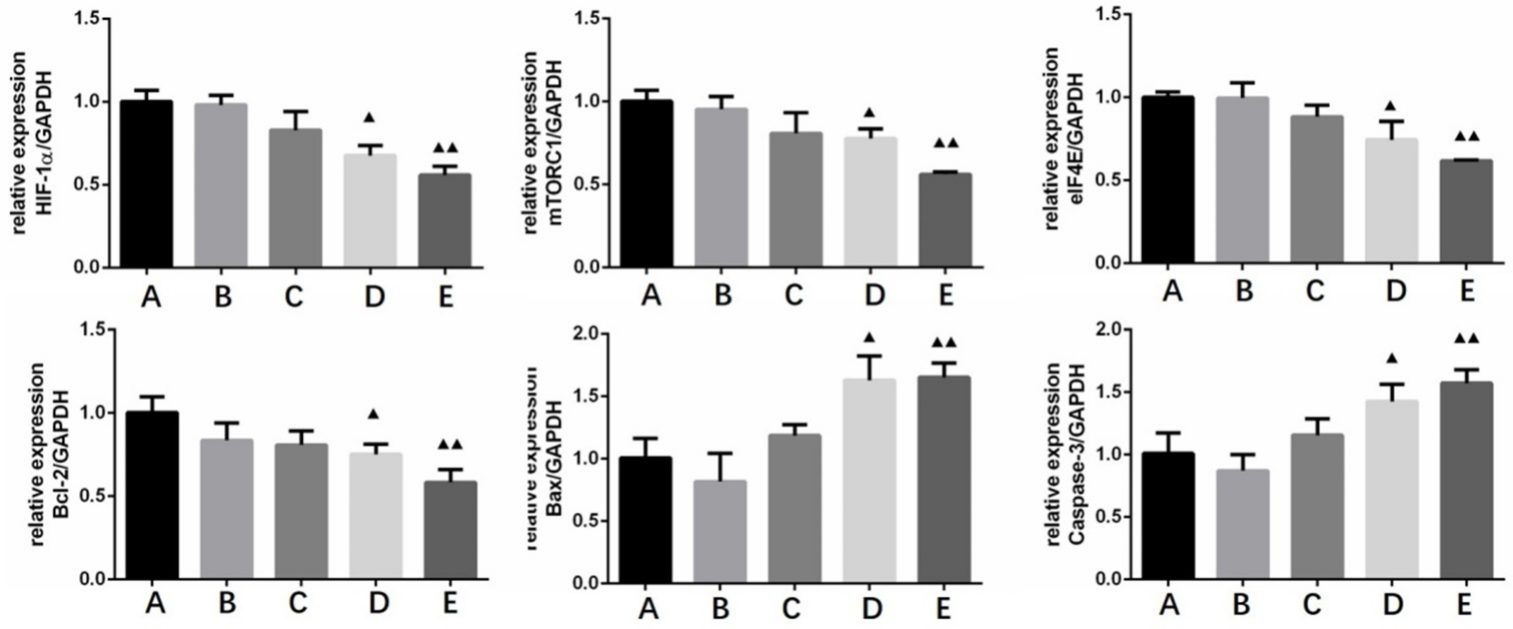
The effect of Borneol on the expression of HIF-1α, mTORC1, eIF4E, Bcl-2, Bax and Caspase-3 in human glioma cells by qRT-PCR. A. Control B. Borneol 10 µg/ml C. Borneol 20 µg/ml D. Borneol 40 µg/ml E. Borneol 80 µg/ml comparison with the control group, ^▲^ P<0.05, ^▲▲^ P <0.01.

HIF-1 α, mTORC1, eIF4E and Bcl-2 were treated with four different concentrations (10, 20, 40, 80 μg/ml) of borneol for 24 hours MRNA expression level showed a downward trend, there was a certain concentration dependence, the higher the borneol concentration, the lower the mRNA expression level; Bax, caspase-3 mRNA expression level showed an upward trend in each group of cells, and there was a concentration dependence, the higher the borneol concentration, the higher the mRNA expression level (Figure 8). When the borneol concentration reached 40 μg/ml, there was a significant difference between the two groups (P < 0.05 or P < 0.01).

### The effects of Borneol on the expression of HIF-1α, mTORC1, eIF4E, p-eif4e, Bcl-2, Bax and Caspase-3 in human glioma cells by Western blot analysis

**Figure 9:**
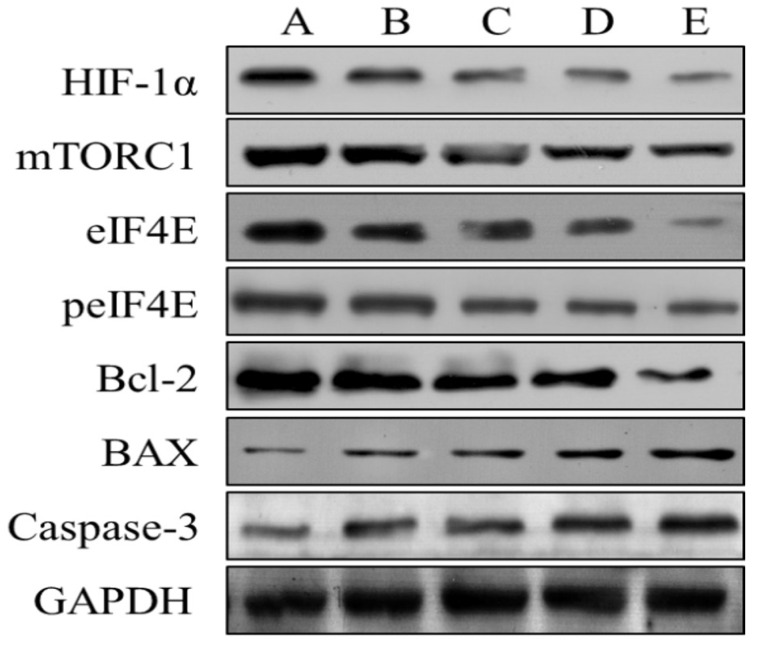
The effects of Borneol on the expression of HIF-1 α, mTORC1, eIF4E, p-eif4e, Bcl-2, Bax and Caspase-3 in human glioma cells by Western blot analysis. A. Control B. Borneol 10 µg/ml C. Borneol 20 µg/ml D. Borneol 40 µg/ml E. Borneol 80 µg/ml.

After four different concentrations (10, 20, 40, 80 μg/ml) of borneol were treated for 24 hours, the expression of HIF-1 α, mTORC1, eIF4E, p-eif4e and bcl-2 protein showed a downward trend, and there was a concentration dependence. The higher the concentration of borneol, the lower the protein expression level; the expression of Bax and caspase-3 protein showed an upward trend, and there was a concentration dependence, borneol concentration showed a downward trend The higher the expression level, the higher the protein expression level (Figure 9). When the borneol concentration reached 40 μg/ml, there was a significant difference between the two groups (P < 0.05 or P < 0.01).

### The effects of Borneol on the expression of Bcl-2, Bax and Caspase-3 in human glioma cells Transfected with HIF-1α Overexpression Plasmid by Western blot analysis

**Figure 10:**
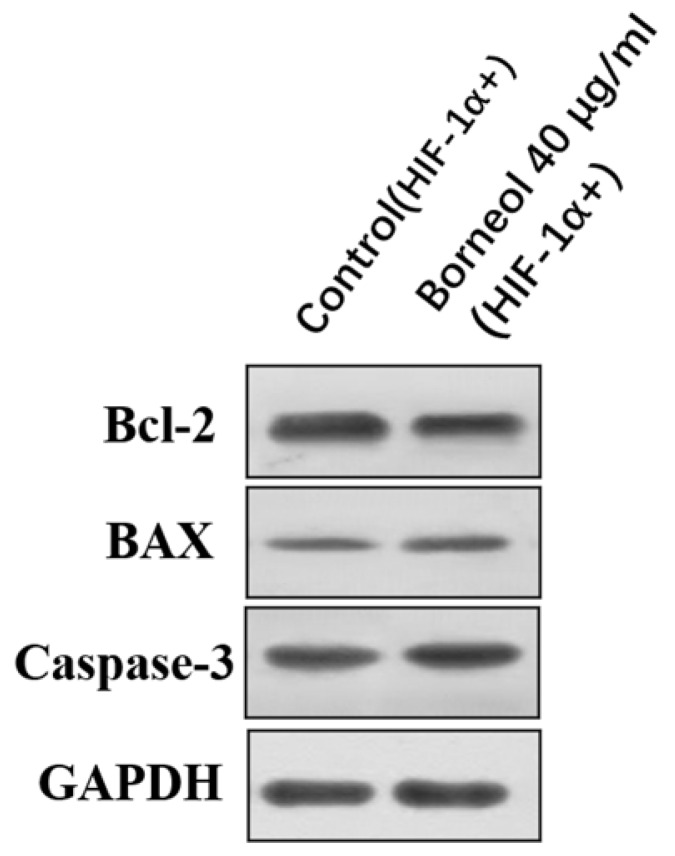
The effects of Borneol on the expression of Bcl-2, Bax and Caspase-3 in human glioma cells transfected with HIF-1α Overexpression Plasmid by Western blot analysis.

As shown in Figure 10, compared with the control group, the expression of bcl-2 protein didn't show an obvious downward trend, and also the expression of Bax and caspase-3 protein didn't show a significant upward trend (P > 0.05).

### Regulation of HIF-1 α expression by silencing mTORC1/eIF4E in human primary glioma cells

**Figure 11:**
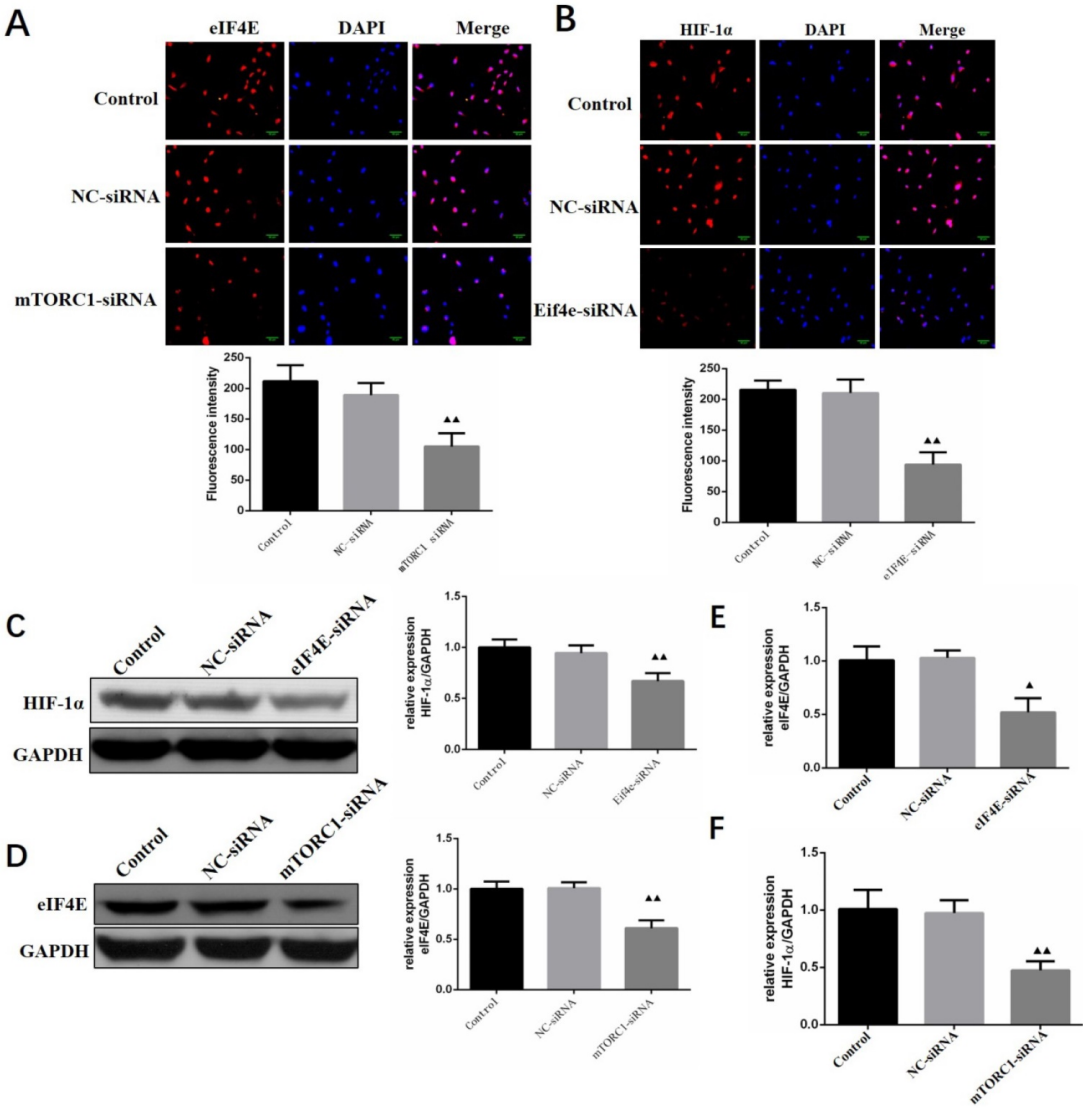
** Regulation of HIF-1α expression by silencing mTORC1 / eIF4E in human primary glioma cells.** A. EIF4E expression in cells of each group (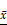 ±S, n=3) comparison with the control group, ^▲^ P<0.05, ^▲▲^ P <0.01; B. Expression of HIF-1α in cells of each group (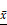 ±S, n=3) comparison with the control group, ^▲^ P<0.05, ^▲▲^ P <0.01; C. Expression level of eIF4E protein in each group after mTORC1 siRNA transfection (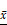 ±S, n=3) comparison with the control group, ^▲^ *P* <0.05, ^▲▲^ *P* <0.01; D. Expression level of eIF4E mRNA in each group after mTORC1 siRNA transfection (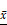 ±S, n=3) comparison with the control group, ^▲^ *P* <0.05, ^▲▲^ *P* <0.01; E. Expression of HIF-1α protein in each group after eIF4E siRNA transfection (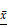 ±S, n=3) comparison with the control group, ^▲^ *P* <0.05, ^▲▲^ *P* <0.01; F. Expression of HIF-1α mRNA in each group after eIF4E siRNA transfection (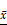 ±S, n=3) comparison with the control group, ^▲^ *P* <0.05, ^▲▲^ *P* <0.01.

The expression levels of eIF4E and HIF-1 α in primary human glioma cells transfected with mTORC1 siRNA were detected by immunofluorescence. As can be seen in Figure 11A, the expression of eIF4E in mTORC1 siRNA transfected cells was significantly lower than that in the control group (P < 0.01). In Figure 11B, it is visible that the expression of HIF-1 α in the cells transfected with eIF4E siRNA was significantly lower than that in the control group (P < 0.01). Western blot results revealed significantly lower expression of eIF4E in mTORC1-siRNA group than that in the control group (P < 0.01). We established that the protein expression level of HIF-1 α in the eIF4E siRNA group was significantly (P < 0.01) lower than that in the control group (Figure 11D). The expression level of eIF4E mRNA in the mtorc1-sirna group significantly decreased (P < 0.05, compared with the control group) after the transfection of human glioma cells with mTORC1 siRNA by qPCR (Figure 11E). Additionally, we found that the expression level of HIF-1 α mRNA in the eIF4E siRNA group was significantly lower (P < 0.01) than that in the control group (Figure 11F). These results indicated that mTORC1/eIF4E silencing downregulated the expression of HIF-1α.

### Correlation analysis and Prognostic analysis

**Figure 12:**
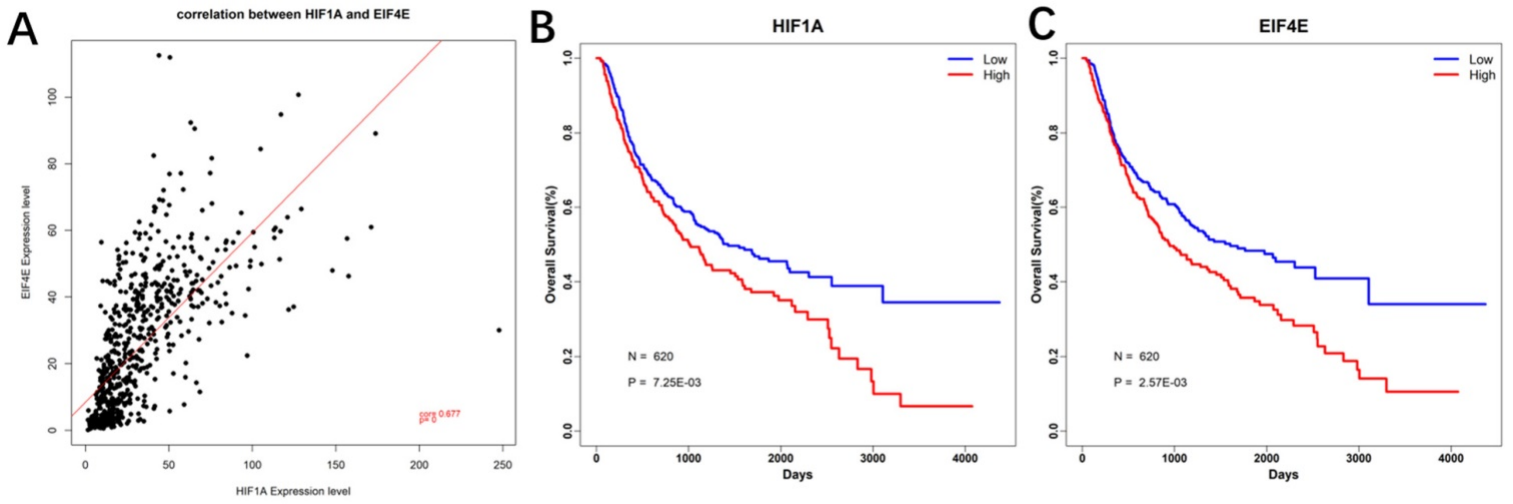
** Correlation and survival curve of HIF-1 α and eIF4E base on CGGA database.** A. Correlation between HIF-1α and eIF4E mRNA expression; B. Survival curve of HIF-1α base on CGGA database; C. Survival curve of eIF4E base on CGGA database.

Based on the expression profile data of HIF-1a mRNA and EIF4E mRNA in GBM, pierce correlation analysis was performed with R software. Further, based on the expression profile data of HIF-1a mRNA and EIF4E mRNA in GBM, patients data were divided into two groups according to their mean values, it showed that there was a positive correlation between the expression of eIF4E and HIF-1 α (P < 0.05) (Figure 12A). At the same time, the prognostic analysis was performed in combination with the clinical information of GBM. The genes were plotted for survival curves, as shown in Figure 12B and 12C. In patients with high expression of eIF4E and HIF-1α had poor survival (P<0.001).

## Discussion

High-grade gliomas (WHO III and IV glioblastomas) are the most common intracranial malignancies in adults. These tumors do not have an intact envelope, grow invasively and are not clearly distinguishable from normal brain tissue. Hence their remove complete surgical removal is difficult, and postoperative radiotherapy and chemotherapy are important adjuvant treatment methods which can significantly improve patient survival 23, 24. However, clinically, the desired effects of postoperative radiotherapy or chemotherapy are often difficult to achieve mainly due to the frequent development of tolerance to radiation or chemotherapy in tumor cells.

Previous research has shown that borneol can open the blood-brain tumor barrier by regulating the expression of TJPs protein 25. In addition, studies have revealed the role of borneol as a new type of chemotherapy sensitizer inducing mitochondrial dysfunction and oxidative damage, which promotes the anticancer effect of temozolomide on human glioma cells 26. However, it is still unclear whether borneol itself could exert an apoptosis effect on malignant gliomas. Therefore, in this study, we evaluated the inhibitory effect of borneol on SD rat brain C6 glioma transplantation model and human primary glioma cell proliferation. Results obtained confirm that borneol could inhibit the proliferation of malignant glioma to some extent. Further, we found that borneol induced human primary glioma cell apoptosis with concentration dependence, but the effect was reversed after transfection of HIF-1 overexpression plasmid. Li Feifei 27 reported that borneol (Natural Borneol) can cause apoptosis of human nasopharyngeal carcinoma, squamous cell carcinoma, adenocarcinoma and breast cancer cells. Apoptosis is a cascade of active cell death process, which is regulated by many genes and closely related to Caspase-3 family and BCL-2 family. The interaction of apoptosis, proliferation and anti-apoptosis of tumor cells determines the degree of malignant progression of tumor biological behavior. Therefore, this study further evaluated the effect of borneol on apoptosis-related regulatory factors. We found that borneol could downregulate the expression of Bcl-2 and upregulation the expression of Bax and caspase-3, borneol could downregulate the expression of Bcl-2 and upregulation the expression of Bax and caspase-3, similarly, the effect was also reversed after transfection of HIF-1 overexpression plasmid, suggesting that the apoptosis effect induced by borneol in human primary cultured glioma cells is mediated via HIF-1α.

The occurrence of hypoxia in the tumor microenvironment changes the transcriptional activity of some genes in tumor cells. The changes in the expression products of tumor cells lead to the adaptation of tumor cells to the hypoxic microenvironment, promoting tumor growth, angiogenesis, metastasis, apoptosis impairment. The transcription factor HIF-1 plays a central role in this process. The HIF-1α gene is the rate-limiting factor of HIF-1 that determines the activity of HIF-1^28^, ^29^. Moreover, the bioinformatics analysis of correlation between HIF-1α and apoptosis-related factors based on CGGA database showed that there was a positive correlation between the expression of eIF4E and HIF-1 α (P < 0.05), and in patients with high expression of eIF4E and HIF-1α had poor survival and prognosis (P<0.001).

Then, we found that eIF4E siRNA suppressed the expression of HIF-1α, whereas the expression of eIF4E was inhibited by the mTORC1 siRNA, suggesting that the mTORC1/eIF4E pathway participates in regulating the expression of HIF-1α in primary human glioma culture cells under hypoxia. It hypoxia has been shown to promote the migration and invasion of malignant glioma cells, which can be achieved by activating PI3K / Akt / mTOR / HIF-1 α pathway 30. Moreover, evidence revealed that borneol enhanced paclitaxel-induced apoptosis of human esophageal squamous cell carcinoma (ESCC) cells by inhibiting the activity of PI3K / Akt 31. In order to clarify the exact mechanism of borneol induced apoptosis in malignant glioma, this study further evaluated the effect of Borneol on HIF-1α, mTORC1, eIF4E expression. Both in SD rat brain C6 glioma transplantation model and human primary glioma cell model, results showed that borneol could inhibit the expression of mTORC1/eIF4E/HIF-1a pathway, implying that borneol could induce primary glioma cells apoptosis by suppressing HIF-1 α expression via the mTORC1 / eIF4E pathway.

In conclusion, the findings of the present study suggest that HIF-1α may be a key factor in apoptosis of glioma cells, and mTORC1/eIF4E pathway is involved in the HIF-1a expression regulation by borneol in malignant glioma. Our results not only reveal the target and molecular mechanism and action of borneol leading to promote apoptosis in glioma cells, but also provide experimental basis and theoretical support for the clinical application of borneol.

## Figures and Tables

**Table 1:** Final tumor volume of rats in each group (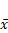
±s, n=6)

	Tumor volume (mm^3^)	Inhibition rate (%)
Model Group	53.99	
Low dose group of Borneol (4 mg/kg)	33.03*	34.50
Medium dose group of Borneol (8 mg/kg)	30.39**	42.25
High dose group of Borneol (16 mg/kg)	24.05**	54.26

Compare with model group: *P <0.05, **P <0.01.

**Table 2:** The effect of Borneol on the expression of HIF-1 α protein in brain glioma of C6 rats (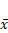
±s, n=6)

Group	Area	IOD
Model Group	129894.0±27834.9	7614.2±1761.8
Low dose group of Borneol(4 mg/kg)	89446.2±21464.8*	4510.0±1252.8*
Medium dose group of Borneol (8 mg/kg)	72740.5±18369.5**	4043.3±1132.0**
High dose group of Borneol(16 mg/kg)	50668.7±12418.1**	2694.2±753.6**

Compare with model group: *P <0.05, **P <0.01.

**Table 3:** Effect of Borneol on the expression of mTORC1 protein in brain glioma of C6 rats (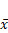
±s, n=6)

Group	Area	IOD
Model Group	135075.3±39024.2	22351.7±7236.9
Low dose group of Borneol(4 mg/kg)	105222.5±41448.5	16569.1±7716.7
Medium dose group of Borneol (8 mg/kg)	89601.7±20803.4*	13659.4±3868.9*
High dose group of Borneol(16 mg/kg)	47369.5±13712.6**	6002.6±2674.9**

Compare with model group: *P <0.05, **P <0.01.

**Table 4:** Effect of Borneol on the expression of eIF4E protein in C6 rat brain glioma model (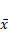
±s, n=6)

Group	Area	IOD
Model Group	152238±17344.3	13570.6±1601.3
Low dose group of Borneol (4 mg/kg)	109643.8±22019.1**	9636.1±2035.3**
Medium dose group of Borneol (8 mg/kg)	82896.3±19349.0**	7264.8±1772.9**
High dose group of Borneol (16 mg/kg)	42909.2±15126.7**	3620.4±1355.6**

Compare with model group: *P <0.05, **P <0.01.
