# Borneol promotes apoptosis of Human Glioma Cells through regulating HIF-1a expression via mTORC1/eIF4E pathway: Erratum

**DOI:** 10.7150/jca.80450

**Published:** 2022-12-30

**Authors:** Zeng Wang, Qinglin Li, Liang Xia, Xia Li, Caixing Sun, Qiong Wang, Xinjun Cai, Guonong Yang

**Affiliations:** 1 Pharmacy Department, Institute of Cancer and Basic Medicine (ICBM), Chinese Academy of Sciences; Cancer Hospital of the University of Chinese Academy of Sciences; Zhejiang Cancer Hospital, Hangzhou 310022, People's Republic of China.; 2 Neurotumor surgery department, Institute of Cancer and Basic Medicine (ICBM), Chinese Academy of Sciences; Cancer Hospital of the University of Chinese Academy of Sciences; Zhejiang Cancer Hospital, Hangzhou 310022, People's Republic of China.; 3 Cancer Institute department, Institute of Cancer and Basic Medicine (ICBM), Chinese Academy of Sciences; Cancer Hospital of the University of Chinese Academy of Sciences; Zhejiang Cancer Hospital, Hangzhou 310022, People's Republic of China.; 4 Department of pharmacy, ZheJiang Chinese Medicine and Western Medicine Integrated Hospital, 310003, Hangzhou, Zhejiang, P. R. China.

Recently, we conducted an examination of our published articles and found that a representative picture (Figure 6E) was incorrect, and the error was made during the assembly of the picture. Below is the corrected figure 6.

## Figures and Tables

**Figure 6:**
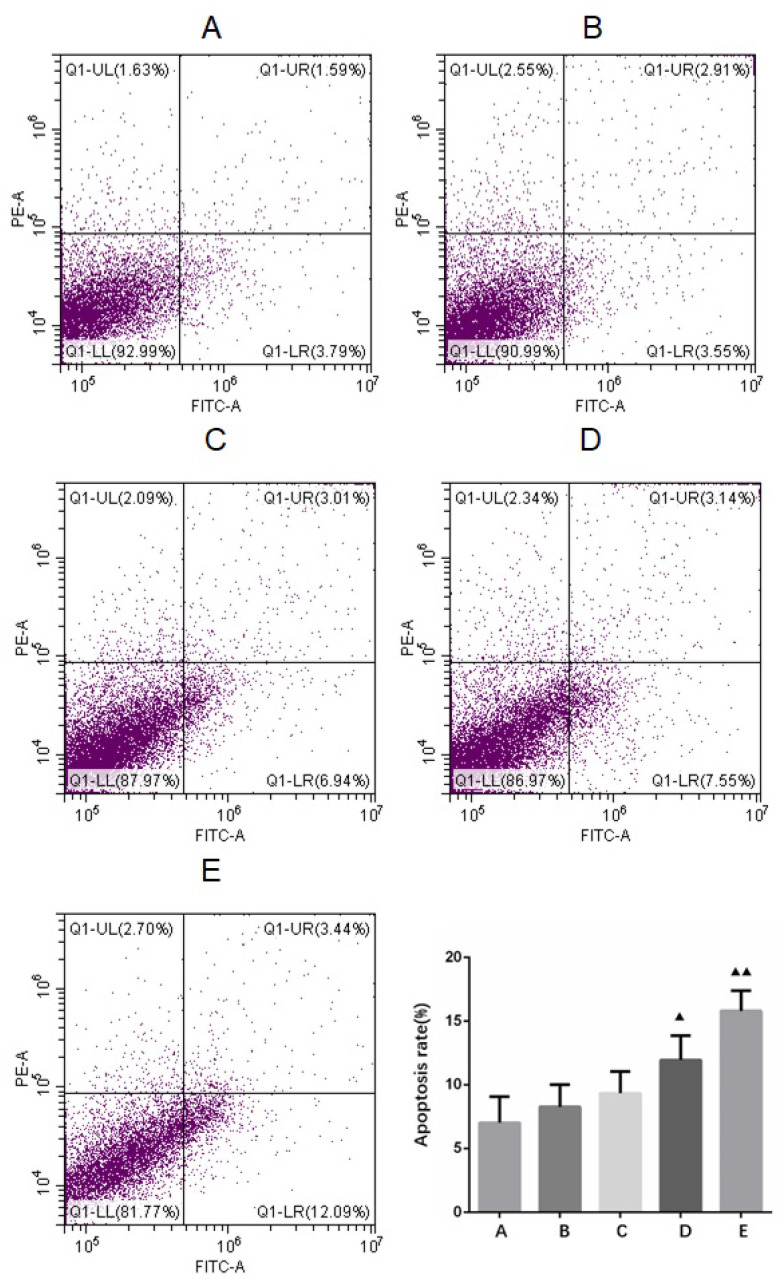
Apoptosis detection in primary cultured human glioma cells. A. Control B. Borneol 10 µg/ml C. Borneol 20 µg/ml D. Borneol 40 µg/ml E. Borneol 80 µg/ml comparison with the control group, ^▲^ P<0.05, ^▲▲^ P <0.01.

